# Matters of the Heart: Cardiotoxicity Related to Target Therapy in Oncogene-Addicted Non-Small Cell Lung Cancer

**DOI:** 10.3390/ijms26020554

**Published:** 2025-01-10

**Authors:** Sara Torresan, Martina Bortolot, Elisa De Carlo, Elisa Bertoli, Brigida Stanzione, Alessandro Del Conte, Michele Spina, Alessandra Bearz

**Affiliations:** 1 Department of Medical Oncology, Centro di Riferimento Oncologico di Aviano (CRO), Istituto di Ricovero e Cura a Carattere Scientifico, 33081 Aviano, Italy; 2 Department of Medicine (DME), University of Udine, 33100 Udine, Italy

**Keywords:** NSCLC, cardiotoxicity, osimertinib, cardioncology, targeted therapy

## Abstract

The treatment of Non Small Cell Lung Cancer (NSCLC) has been revolutionised by the introduction of targeted therapies. With the improvement of response and frequently of overall survival, however, a whole new set of adverse events emerged. In fact, due to the peculiar mechanism of action of each one of the tyrosine kinase inhibitors and other targeted therapies, every drug has its own specific safety profile. In addition, this safety profile could not fully emerge from clinical trials data, as patients in clinical practice usually have more comorbidities and frailties. Cardiotoxicity is a well-known and established adverse event of anti-cancer therapies. However, only recently it has become a central topic for targeted therapies in NSCLC, due to the unknown real range and frequency. Management of this toxicity begins with prevention, and must balance the need of continuing an effective anticancer treatment versus low risk of even fatal events and the preservation of long-term quality of life. The aim of this review is to summarise the current knowledge focusing on currently used targeted therapies in NSCLC.

## 1. Introduction

In the last decade, the introduction of new effective treatment options such as Tyrosine Kinase Inhibitors (TKIs) and Antibody–Drug Conjugates (ADCs) has notably increased survivorship for patients with non-small cell lung cancer (NSCLC) and Actionable Genomic Alterations (AGAs) [1]. However, targeted therapy is not exempted from toxicity and cardiac adverse events (CAEs) frequently occur during cancer treatment.

Cardiotoxicity is specifically defined as any effect of anticancer drugs on the cardiovascular system, but also as an indirect impact via hemodynamic or coagulation changes [2]. It is commonly divided into two types. Type I, characterized by an early onset, is irreversible, dose-dependent and related to myocardial damage; it is typically associated with chemotherapy. Type II cardiotoxicity can have a later onset, is not dose-dependent and is generally reversible; this is typically associated with TKIs [3]. Although the unique mechanism of action of different targeted therapies (TTs) makes it complicated to understand the precise pathway leading to cardiotoxicity, common hypotheses comprise on-target and off-target effects, due to the different selectivity of the various TTs. Involved pathways usually can lead to multiple deleterious vascular effects, mitochondrial dysfunction and ion channel inhibition, all leading to different CAEs [4]. 

Diagnosis of cardiotoxicity can be challenging because of patients’ comorbidities and cancer-related symptoms that can mask underlying conditions. In addition, the grading of CAEs is often subjective. Common Terminology Criteria for Adverse Events (CTCAE) comprises 35 terms for CAEs, ranging from electrical (arrhythmias, QTc prolongation) to functional (Left Ventricular Ejection Fraction (LVEF) decrease and heart failure (HF)) or structural (cardiomyopathies), but all of them are graded according to the presence of not clearly specified symptoms and the need for medical intervention, which is at the physician’s discretion.

Moreover, most of the clinical trials specifically excluded patients with cardiovascular risk factors (e.g., LVEF decrease or QT prolongation at screening) or known cardiovascular disease such as previous myocardial infarction, limiting the understanding of the true extent of the problem. Indeed, patients with cardiovascular underlying disease represent an important percentage of those treated in daily clinical practice [5]; additionally, real-world data are fundamental to understanding the true extent of the problem. NSCLC patients compared to other cancer populations are generally older and have more comorbidities and cardiovascular (CV) risk factors (such as tobacco use and co-medications), elements that could enhance the risk of cardiotoxicity [2,6,7,8]. 

Finally, although the major cardiovascular and oncology societies have developed guidelines to assist clinicians in the early detection, prevention and treatment of cardiotoxicity, some issues remain. Indeed, traditional cardiovascular risk models are insufficient to determine the risk of CAEs, as they do not incorporate the newest cancer therapies and are not specifically designed for cancer patients. 

The aim of this review is to summarise the current knowledge about specific TT-induced CAEs in patients with NSCLC and AGA, underline the open issues in this subgroup and suggest the proper specific management. Please note that not all possible targeted therapies have been included, as we only included the most currently used treatments.

## 2. Cardiotoxicity of Targeted Therapies in NSCLC

### 2.1. EGFR Inhibitors

Endothelial Growth Factor Receptor (EGFR) is a transmembrane tyrosine kinase which activates different signalling pathways that lead to cell proliferation, including Mitogen Acyivated Protein Kinase (MAPK) and Ak strain Trasforming (AKT) cascades [9]. Its mutations are the most frequent targetable alterations in NSCLC, and the currently approved and used drugs are osimertinib, afatinib, amivantamab and lazertinib.

Osimertinib irreversibly binds to the EGFR kinase by targeting the C797 residue in the ATP binding site, obtaining minimal off-target activity [10]. Nonetheless, osimertinib is responsible for a variety of CAEs.

Percentages of CAEs reported for osimertinib in the registrational trials are reported in Table 1. The most relevant CAEs for osimertinib are QTc prolongation and asymptomatic LVEF decrease, globally not frequent. Most CAEs emerged during the first six months of treatment and were usually mild and manageable [11,12]. Treatment interruption was reported to be resolutive, and CAEs did not reappear at the resumption of the drug [13].

Particular attention must be paid when combining osimertinib with chemotherapy, as the incidence of CAEs increases [14]. 

Combined analysis of the 2 phase III randomised trials AURA3 and FLAURA found an increased estimated pooled risk ratio of HF and QTc prolongation of 2.7 (*p* = 0.03) and 2.6 (*p* = 0.003), respectively, for osimertinib versus the comparator arms. Still, HF was rare, occurring in 21 (3.8%) of 558 patients treated with osimertinib in these 2 trials [15].

In the Food and Drug Administration (FDA) pooled safety analysis of 833 patients, cardiomyopathy (HF, congestive HF, pulmonary edema, or decreased ejection fraction) occurred in 1.9% of patients and included 1 (0.1%) fatal event [16].

Real-world data show a greater incidence of cardiac toxicity, presumably because patients with more comorbidities and risk factors were treated than those included in clinical trials. Data from the World Health Organization (WHO) pharmacovigilance database reported that osimertinib was more strongly associated with QT prolongation, HF and supraventricular tachycardia than other targeted therapies [17]. Data from the Food and Drug Administration Adverse Events Reporting System (FAERS) show a relatively higher risk of HF, atrial fibrillation (AF), QT prolongation, myocardial infarction, and pericardial effusion with osimertinib compared to other drugs considered, but similar to other TKIs [18].

A real-world treatment study of 3015 patients reported a 2% incidence of QTc prolongation (none serious), but HF was not assessed [19].

The molecular mechanism underlying osimertinib cardiotoxicity is not fully understood yet, as the EGFR pathway and the on-target and off-target toxicity of osimertinib are complex and intertwined with all other intracellular pathways. For HF, the possible mechanism is the on-target impaired dimerisation with Erbb2, whose role in HF is well known [20]. Notably, in a pooled analysis of patients from FLAURA, AURA3 and the osimertinib expanded access program (EAP), no relationship between systemic exposure of osimertinib or AZ5104 (osimertinib active metabolite) and changes in LVEF was found [21].

In animal models, a reduction in ATP production (due to disruption by osimertinib of the ATP synthase), leads to mitochondrial malfunction and subsequent oxidative stress and myocardiocyte damage [22], as confirmed in cardiac biopsies of patients with CAEs [23,24], hinting at a possible off-target mechanism. 

As regards QT prolongation, this may be due to osimertinib blockage of hERG and various ion channels [25] or the inhibition of the Phosphoinositide 3-kinase (PI3K) pathway, which affects these same ion channels as another possible off-target mechanism [26].

Afatinib is a second-generation EGFR inhibitor which irreversibly inhibits all Erbb family receptors and is nowadays predominantly used for uncommon *EGFR* mutations.

Of the many clinical trials which evaluated this drug, very few CAEs were reported. In particular, the LUX-LUNG7, LUX-LUNG6 and LUX-LUNG2 trials reported no CAEs [27,28]. Clinical trials which reported CAEs are illustrated in Table 1.

A combined analysis from different trials (for a total of 3865 afatinib-treated patients) showed that the adjusted risk for HF was 1.4%, the majority of which were G ≤ 2. A significant decrease in LVEF was seen in only 4.6–6.3% of patients [29].

A phase II study on solid tumours was specifically carried out to determine the cardiac toxicity of afatinib, revealing no prolongation of QTcF for a dose of afatinib of 50 mg [30]. 

As for osimertinib, the targeting of Human Epidermal Growth Factor Receptor 2 (HER2) along with EGFR and PI3K pathway dysregulation with ion channel blockage have been proposed as potential on- and off-target mechanisms for afatinib-induced cardiotoxicity [31,32]. Another possible off-target mechanism is the induction of the endoplasmic reticulum stress pathway, which promotes inflammation (via increased reactive oxygen species (ROS) and peroxidation) leading to myocardiocyte damage in a dose- and time-of-exposure-dependent way, as seen in rat models [33].

Dacomitinib is another second-generation EGFR inhibitor active against all Erbb2 family members. It has not been associated with relevant CAEs in the registrational trial (see Table 1) and real-world experiences did not report new unexpected CAEs [34]. 

Erlotinib and gefitinib are first-generation EGFR inhibitors not usually associated with high incidences of CAEs (see Table 1). However, this could be due to the age when clinical trials with these drugs were performed. In the literature and in the FDA label, in fact, cases of myocardiopathy with erlotinib are reported and attention to QTc interval prolongation is advised [34]. Similarly, for gefitinib no particular cardiac safety concerns emerge from the registrational trials (see Table 1) and none is reported in the drug labels; however, real-world experiences and case reports advise that cardiomyopathy, QTc prolongation and acute coronary syndrome must be taken into account [34,35]. Gefitinib cardiotoxicity in particular has been attributed to a hyperactivation of the platelets, leading to acute coronary syndrome, while interference with the Phosphatase and tensin homolg (PTEN)/Akt pathway and the formation of CYP1A1-induced reactive metabolites could lead to cardiomyopathy as seen in rat models [34]. Particular attention should be paid when gefitinib and erlotinib are administered in combination with antiangiogenic drugs as advised in some guidelines, as thromboembolic risk could be enhanced [36]. 

Amivantamab is a bispecific antibody that binds the extracellular domains of EGFR and Mesenchymal epithelial transition factor receptor (MET). In the CHRYSALIS phase I study, cardiac toxicities ≥G3 were reported in 4 cases (one each of acute coronary syndrome, atrial flutter, cardio-respiratory distress and sudden death) [37]. CAEs for the registrational trials are reported in Table 1.

In the PAPILLON and PALOMA3 trials, no treatment-related CAEs were described [38,39].

Preliminary real-world data did not show CAEs, but cardiac assessment and cardiotoxicity surveillance were unclear, not reported and possibly insufficient [40]. In fact, in the FAERS database, amivantamab-related CAEs constituted 16.5% of all reported AEs. The most frequent were abnormal blood pressure and arrhythmia. The latter had the shortest time to onset (TTO) (0–13 days), while coronary artery disease was reported as late-onset CAEs (median TTO of 570 days). Most CAEs occurred in the first 3 months [41].

Lazertinib is a third-generation irreversible EGFR inhibitor with high selectivity [42]. In addition to the abovementioned clinical trials when combined with amivantamab, no relevant CAEs were highlighted in the clinical trials evaluating lazertinib alone, except for G3 QT prolongation in 2 patients (1%) in the LASER301 trial [43,44] (see Table 1). Preclinical and clinical evaluation revealed little to no physiological effect on electrocardiogram (ECG) [45].

Preliminary real-world data seem to confirm the relative safety of lazertinib’s cardio-toxicity profile [46], specifically for QTc [47]. The paucity of CAEs could be explained by the high selectivity of lazertinib, which results in minimal inhibition of HER2 [46].

**Table 1:** Cardiotoxic events reported in the registrational or most relevant clinical trials for all approved targeted therapies in oncogene-addicted NSCLC. Abbreviations: LVEF: left ventricle ejection fraction; HF: heart failure; NR: not reported; AF: atrial fibrillation; ECG: electrocardiogram.

Drug	Clinical Trial	QTc Prolongation	Hypertension	LVEF Decrease/HF	Arrhythmia	Other Relevant
Osimertinib	AURA3 [11]	2%	NR	5%/3%	NR	NR
	FLAURA [12]	10%	NR	3%/4%	NR	NR
	ADAURA [13]	1%	NR	<1%	1 case (supraventricular)	Cardiomyopathy and miocardial infarction < 1%
	FLAURA2 [14] (data in the combination arm)	NR	NR	6%/2% (2HF G5)	1 case (ventricular arrhythmia)	Cardiomyopathy 7% One episode each of cardiac arrest, coronary stenosis, myocarditis.
Afatinib	LUXLUNG8 [47]	NR	NR	0.3% G3 HF	Only G3 reported AF (0.8%), supraventricular tachycardia (0.5%), sinus tachycardia (0.3%)	G3 palpitations (0.5%), angina pectoris (0.3%)
	LUXLUNG3 [48]	NR	NR	2%	NR	NR
Dacominitnib	ARCHER-1050 [49]	NR	5%	NR	AF 2%	Hypotension 2%
Erlotinib	EURTAC [50]	NR	NR	NR	NR	NR
Gefitinib	NCT01203917 [51]	NR	7.5%	HF 2.8%	NR	NR
Amivantamab	MARIPOSA [52]	NR	NR	NR	NR	Myocardial infarction (0.7%) and sudden death (0.7%)
	MARIPOSA2 [53]	NR	NR	NR	Fatal ventricular fibrillation 0.8% supraventricular tachycardia 2%	NR
Lazertinib	LASER301 [43]	G3 1%	NR	NR	NR	NR
Alectinib	ALEX [54]	NR	NR	NR	NR	NR
	J-ALEX [55]	3%	NR	NR	Bradycardia 1%	NR
	ALESIA [56]	NR	NR	NR	Sinus bradycardia 30%	NR
	ALINA [57]	NR	NR	NR	NR	1.6% myocardial infarction
Brigatinib	ALTA [58]	NR	32%	NR	Bradycardia 13.3%	NR
	ALTA1 [59]	NR	31%	NR	Bradycardia 12%	NR
	ALTA3 [60]	NR	22%	NR	NR	NR
Ceritinib	ASCEND-4 [61]	12%	NR	NR	NR	1 fatal myocardial infarction
	ASCEND-5 [62]	11.1%	0.9% (0.9% hypertensive crisis)		AF 2.7%, Atrial flutter 0.9%, ECG T-wave inversion 0.9%	Myocardial ischemia 1.8%, angina pectoris 0.9%
Lorlatinib	CROWN [63]	NR	18%	HF 1%	Sinus bradycardia 4% PR prolongation 14% Atrioventricular block 1%	Hypertriglyceridemia (64%) and hypercholesterolemia (70%)
	B7461001 [64]	NR	1.5%	2%	NR	Hypercholesterolemia 81%, hypertriglyceridemia 61%
Crizotinib	PROFILE-1007 [65]	4% G3–4	NR	NR	Bradycardia 3%, one fatal supraventricular arrythmia	NR
	PROFILE-1014 [66]	2% G3–4	NR	2.7%/2.3%	Bradycardia 14%	NR
Entrectinib	ALKA-372-001, STARTRK-1, and STARTRK-2 [67]	2%	0.9%	1.3%/2.6%	NR	Hypertriglyceridemia 2.6%, hypotension 7.9%, acute coronary syndrome 0.4%, myocarditis 0.4%
Repotrectinib	TRIDENT-1 [68]	NR	NR	NR	NR	NR
Dabrafenib and trametinib	BRF113928 [69]	NR	NR	9%	14%	Cardiac arrest 3%
Encorafenib and binimetinib	PHAROS [70]	NR	NR	NR	NR	NR
Adagrasib	KRYSTAL-1 [71]	20%	NR	4.3%/3.4%	AF 2.6%	Hypotension 11.2%
Sotorasib	CODEBREAK-100 [72]	2%	NR	HF 2.4%	Sinus tachycardia and tachycardia (2.4% each), other arrhythmias 0.8%	Hypotension (4%), hypercholesterolemia (3.2%), myocardial infacrtion 0.8%
Pralsetinib	ARROW [73]	NR	25%	NR	NR	NR
Selpercatinib	LIBRETTO-431 [74]	20%	48%	NR	NR	NR
Capmatinib	GEOMETRY [75]	NR	NR	NR	NR	Cardiac arrest (1 case)
Tepotinib	VISION [76]	NR	NR	NR	NR	NR
Trastuzumab deruxtecan	DESTINY-LUNG01 [77]	NR	NR	NR	NR	NR
Larotrectinib	Pooled analisys [78]	<3%	10%	<3%	AF < 3%, bradycardia <3%	Hypotension 7%

### 2.2. ALK Inhibitors

The anaplastic lymphoma kinase (ALK) is a transmembrane kinase involved in various signalling pathways, including the MAPK and PIK3 ones [79]. *ALK* rearrangements account for approximately 4–5% of AGAs in NSCLC [80]. Approved TKIs in this setting are alectinib, brigatinib, ceritinib and lorlatinib.

The most common CAE of ALK inhibitors is bradycardia, which appears in the first 2 months [81]. FAERS reported a higher incidence of cardiac disorders as a whole for crizotinib and lorlatinib [81], and more reports of arrhythmias for alectinib and crizotinib [82]. Data from the WHO pharmacovigilance database showed higher odds of conduction disease (reporting OR (ROR) = 12.95, 99% CI: 10.14–16.55) and QT prolongation (ROR = 5.16, 99% CI: 3.92–6.81) for ALK inhibitors than for other TTs [17]. In the same database, higher odds for hypertension were identified for brigatinib (ROR, 3.9; 95% CI 2.88–5.20) and for HF with alectinib (ROR, 2.2; 95% CI 1.60–2.90), crizotinib (ROR, 2.1; 95% CI 1.72–2.48), and lorlatinib (ROR, 2.0; 95% CI 1.27–3.23) [83]. 

An analysis of 2855 patients revealed that crizotinib and alectinib were more likely to cause arrhythmias while crizotinib and lorlatinib were more likely to cause blood pressure abnormalities [84]. In another pooled analysis (*n* = 1737), bradycardia incidence was 8% and no differences were noted between the different TKIs [85]. Patients with pre-therapy heart rate (HR) < 60 bpm had a higher risk (hazard ratio [HR] 6.7, 95% confidence interval (CI) 1.6–28.5, *p* = 0.011), especially if they received chemotherapy prior to ALK inhibitors (HR 7.2, 95% CI 2.0–26.1, *p* = 0.003) [86]. 

CAEs for each TKI reported in the most relevant clinical trials are summarised in Table 1.

Alectinib is currently approved as first-line treatment in *ALK*-rearranged NSCLC.

In the pooled safety analysis on the FDA label (including NP28761, NP28673, ALEX and ALINA trials), bradycardia occurred in 11% of patients, and 20% of patients had post-dose HR < 50 bpm, but no clinically relevant QT prolongation was observed.

In a retrospective analysis of two clinical studies, no clinically significant effect of alectinib on ECG was found, except for an asymptomatic decrease in the HR by 11–13 bpm [87].

In real-world studies, the most common arrhythmia reported for alectinib is bradyarrhythmia [88], intended both as ECG-documented or as HR < 50 bpm at least once during treatment [89]. In a real-world cohort of 53 patients, LVEF did not significantly decrease, but 42% of patients developed bradycardia, mostly asymptomatic (except for one case that required a pacemaker) [90]. In another retrospective cohort (*n* = 93), bradycardia was recorded in 50.54% of patients, with a median change from baseline HR of 11 bpm, and was reversible upon reduction or withdrawal of alectinib. Baseline low HR (OR 0.86, 95% CI 0.79–0.93, *p* < 0.001) and history of hypertension (OR 13.71, 95% CI 2.49–76.38, *p* = 0.003) were significantly associated with bradycardia occurrence on multivariate analysis [91].

Brigatinib is another TKI inhibitor with activity against not only *ALK*, but also Ros Protoncogene 1 (*ROS1*), insulin-like growth factor-1 receptor (*IGF-1R*), and *FLT-3* as well as *EGFR* deletion and point mutations. Its most frequent toxicity, along with bradycardia, is hypertension [92].

In the early phase I–II trial, only a few CAEs were reported: 2 cases each of AF and supraventricular tachycardia and 1 episode each of congestive HF, unstable angina unstable and tachycardia in 137 patients [93]. No relevant QTc prolongation was found, independently of dosage [92].

Hypertension with brigatinib treatment appeared dose-related, with a median TTO of 2.1–5.8 months [94]. However, the mechanisms of hypertension related to brigatinib remain unclear.

Ceritinib is another approved ALK inhibitor, whose registrational trials did not report high incidence of CAEs (see Table 1). Nonetheless, the FDA label requires close monitoring for QTc prolongation and bradycardia. In fact, in the overall analyses comprising all clinical trials involving ceritinib, 1.3% of patients had prolonged QTc, in a concentration-dependent mechanism, leading to discontinuation in 0.2% of cases. Similarly, bradycardia was noted in 1.1% overall but no discontinuation was required. Studies from the FAERS database report for ceritinib a previously unseen risk of myocarditis and pericarditis [81].

Lorlatinib is a kinase inhibitor with in vitro activity against ALK and ROS1 as well as other targets. 

Differently from other ALK inhibitors, the most frequent G3–4 AEs were hypertriglyceridemia (20%) and hypercholesterolemia (16%) (see Table 1). Notably, a portion of patients had altered hypercholesterolemia and hypertriglyceridemia at baseline, and these patients experienced the most serious hyperlipidemia [63].

At least one lipid-lowering agent was needed for most patients (81.0%) who received lorlatinib, and 22.1% and 30.8% required two or more agents for hypercholesterolemia and hypertriglyceridemia, respectively [95].

In the FDA label safety population (*n* = 476, 327 from B7461001 and 149 from CROWN), G3–4 hypercholesterolemia occurred in 18% of patients, and G3–4 hypertriglyceridemia in 19%, with a median TTO of 15 days. Hyperlipidemia required temporary discontinuation in 11% of patients and dose reduction in 4%. AV block developed in 1.9% of patients (0.2% G3 requiring pacemaker). Hypertension occurred in 13% of patients (G3–4 6%), with a median TTO of 6.4 months, and a temporary discontinuation rate of 2.3%.

The FAERS database for lorlatinib reports other CAEs, such as HF (*n* = 34), pericardial effusion (*n* = 33) and QTc prolongation (*n* = 26) [96].

Many of the cardiotoxicity mechanisms of ALK inhibitors remain unknown; however, some hypotheses can be considered. For example, ALK inhibitors are known to possibly cause hypogonadism as an off-target effect [97]. The subsequent reduction in testosterone levels could contribute to the observed hyperlipidemia.

Inhibition of funny current in the sinoatrial node has been proposed as the underlying cause of bradycardia [98]. For QT prolongation, the generally recognised mechanism via PI3K/Akt pathway and ion-channel off-target alterations could be applied [99]. As for all TKIs, drug–drug interactions play a fundamental role in cardiovascular risk; however, alectinib has weaker bonds with CYP3A4 metabolism and could therefore have fewer interactions [100].

### 2.3. ROS-1 Inhibitors

ROS-1, a transmembrane tyrosine kinase receptor similar to ALK, activates the MAPK and PI3K signalling pathways [101]. *ROS-1* fusions account for roughly 2% of AGAs in NSCLC [80] and are currently targeted with the oral inhibitors crizotinib, entrectinib and repotrectinib.

Crizotinib is a small-molecule TKI targeting ALK, MET and ROS1 which is seldomly associated with severe CAEs (see Table 1).

In the FDA label safety pooled analysis, 5% of patients had significant (≥60 ms) QTcF prolongation from baseline, which appeared to be concentration-dependent. Bradycardia occurred in 13% of the patients, and G3 syncope was reported in 2.4% of patients.

In the real-world setting, among ALK and ROS1 inhibitors, crizotinib demonstrated a higher risk for conduction disease (ROR = 1.75, 99% CI: 1.30–2.36) and QT prolongation (ROR = 1.91, 99% CI: 1.22–3.00) [17,102].

The WHO safety database recorded an even higher risk for QTc prolongation (ROR, 5.0; 95% CI 3.72–6.77) [83]. In the FAERS dataset, the most frequently reported CAEs for crizotinib were bradyarrhythmia, HF, and pericardial disease (23.2%, 22.7% and 16.7% of all the CAEs, respectively). However, it is important to notice that other CAEs, such as AF and myocardial infarction, were described [103,104].

The underlying mechanism related to crizotinib cardiotoxicity has been evaluated in vitro and in vivo. Off-target mechanism hypotheses comprehend the antagonism of L-calcium channels and the alterations of the function of sinoatrial and atrioventricular nodes with inhibition of the funny current [98,104]. In vitro experiments showed the relations of crizotinib exposure to ROS increase, cholesterol accumulation, and inhibition of ion channels [105]. 

In vivo models showed ventricular dysfunction and myocardial injury via apoptosis and mitochondrial dysfunction through the MAPK pathway [106]. These effects seem to be dose-dependent [98,104].

Entrectinib is a new-generation ROS-1 inhibitor which targets neurotrophic tyrosine receptor kinase (NTRK) as well.

CAEs related to this drug reported in the registrational trials are reported in Table 1.

In the integrated analysis of the clinical trials of entrectinib in NSCLC (ALKA-372-001, STARTRK-1, and STARTRK-2), CAEs were the most frequent reason for entrectinib discontinuation [67]. However, it is worth noting that in these clinical trials, routine ECG or other cardiologic exams were not performed.

In the most recent phase II–III BFAST trial, G3-5 HF was reported in 3.6% of patients, while data on hypotension are currently not available [107].

In the FDA label safety pooled analysis (which considered STARTRK-NG in addition to the aforementioned studies), CAEs incidence was higher (hypotension 18%, 2.8% G3 and QTc prolongation 3.1%). Notably, one patient developed myocarditis, and there are few case reports describing this CAE as well [108,109]. 

Peculiarly, a phenomenon mimicking Brugada syndrome has been associated with entrectinib in more than one case report [110,111].

Other real-world data reported from FAERS seem to confirm the low rate of HF and QT prolongation associated with entrectinib [112].

No studies were conducted to explain entrectinib-related cardiotoxicity. However, given the overlapping with the mechanisms of crizotinib, similar pathways could be involved.

Repotrectinib is the newest ROS1 and NTRK inhibitor, and few data are available. In the TRIDENT-1 trial, fatal cardiac events were reported, but none was attributed to the drug. In the same study, no relevant QT prolongation or other ECG abnormalities were described (see Table 1) [68].

### 2.4. BRAF and MEK Inhibitors

V-Raf Murine Sarcoma Viral Oncogene Homolog B (*BRAF*) encodes for a serine/threonine protein kinase that plays an essential role in regulating the (MAPK)/Extracellular signal-regulated Kinase (ERK) signalling pathway, which affects cell division, differentiation, and secretion [113]. Mutations in the *BRAF* gene, most commonly V600 mutations, are identified in approximately 2% of NSCLC [80]. The standard treatment for patients with this mutation is a combination therapy with BRAF and Mitogen Activated Protein Kinase (MEK) inhibitors. Two regimens have been approved in NSCLC: dabrafenib plus trametinib and, more recently, encorafenib plus binimetinib. 

Dabrafenib was the first BRAF inhibitor to reach the market, while trametinib is a reversible inhibitor of MEK1 and MEK2 kinase activity. Data from the registrational trial of their combination in NSCLC are reported in Table 1. Notably, in the real-world setting, trametinib was also associated with AF [88]. No specific CAEs were reported in the PHAROS or IFCT-1904 ENCO-BRAF trials regarding the combination of encorafenib and binimetinib [70,114].

An increased risk of cardiotoxicity, especially LVEF reduction (5–11%), hypertension (11–30%), or QT prolongation (0–5%), was highlighted with BRAF and MEK inhibitors treatment [115]. Data from the WHO pharmacovigilance database reported higher odds of HF for dabrafenib (ROR = 2.24, 99% CI: 1.86–2.70) and trametinib (ROR = 2.44, 99% CI: 2.03–2.92) compared to other targeted therapies, especially when they are combined together [17]. In another meta-analysis of 5 randomised clinical trials (*n* = 2317) BRAF/MEK inhibitors therapy was associated with an increased risk of LVEF decrease (RR, 3.72; 95% CI, 1.74–7.94; *p* < 0.001) and arterial hypertension (RR, 1.49; 95% CI, 1.12–1.97; *p* = 0.005) compared to BRAF inhibitor monotherapy [116]. 

In the FAERS dataset for BRAF/MEK inhibitors as a group, 7.4% of all reports were for CAEs. Combination therapy was associated with a higher risk of HF (ROR = 1.62 CI = 1.14–2.30; *p* = 0.007) and hypertension (ROR = 1.75 CI = 1.12–2.89; *p* = 0.02), and in general of CAEs during the first six months of treatment (26.2% vs. 16.7%) compared to monotherapy [117]. There was no difference between the combination of dabrafenib/trametinib and vemurafenib/cobimetinib in terms of CAEs [118]. BRAFi/MEKi-induced cardiotoxicity is often reversible upon treatment discontinuation or dose adaptation [107].

The MAPK pathway has a well recognised role in cardiomyocyte repair, survival and proliferation [115,119].

In vitro and in vivo assays demonstrated that the activation of MEK1 induces cardiac hypertrophy and cardiomyopathy [120,121,122]. This on-target mechanism is proven in humans by the existence of genetic syndromes due to mutations in this pathway, which include hypertrophic cardiomyopathies as clinical manifestations [123,124].

The possible cardiotoxic mechanism of BRAF and MEK inhibitors, hence, is related to the suppression of ERK1/2, but also off-target mechanisms affecting PI3K/AKT and Janus tyrosine kinase (JAK)/Signal Transducers and Activators of Transcription (STAT) activation, oxidative stress, and myofibrillar degeneration are known or hypothesised [121,122,125]. MEK and ERK signalling are also involved in the renin–angiotensin system regulation and in the proliferation of smooth muscle cells, which could explain hypertension [106]. Another mechanism for hypertension is the ERK-induced overexpression of CD47, which dysregulates nitric oxide control on vascular tone and subsequently blood pressure [126]. 

### 2.5. KRAS Inhibitors

Kirsten rat sarcoma viral oncogene homologue (KRAS) is the most frequently mutated tyrosine kinase in NSCLC (~30%). Unfortunately, at present, only *KRASG12C* mutations are druggable, and available TKIs are adagrasib and sotorasib. 

Adagrasib is an irreversible inhibitor of KRAS G12C that belongs to the RAS GTPase family. It is associated with QTc prolongation and hypotension (see Table 1); however, data from registrational trials evaluating adagrasib in combination with other drugs are not evaluable [71].

Sotorasib is another G12C selective KRAS inhibitor which is associated with a low incidence of CAEs (see Table 1) [72].

Real-world cohorts showed no evidence of CAEs [127], and in vitro and in vivo safety studies did not highlight on- or off-target cardiotoxicity [128], confirming the relative cardio-safety of these drugs.

### 2.6. RET Inhibitors

Rearranged during transfection (RET) is a tyrosine kinase receptor whose activating *RET* alterations elicit downstream signalling through PI3K/AKT, MAPK, and PLCγ pathways [129]. *RET* fusions occur in approximately 2% of NSCLCs and are targeted by two currently approved TKIs, pralsetinib and selpercatinib.

Pralsetinib is an ATP-competitive inhibitor that inhibits both wild-type and mutated or rearranged RET, and has shown activity against Vascular Endothelial Growth Factor Receptor (VEGFR) 2 [129]. 

In the registrational ARROW trial, the most common CAE was hypertension, both in the treatment-naive population and in the pre-treated subgroup (see Table 1). Overall, hypertension caused dose interruption in 8% of patients and dose reduction in 4.8% [73]. Case reports deriving from clinical practice described events of HF [130]. 

Selpercatinib is another TKI with the same target and similar selectivity to pralsetinib [129]. In the LIBRETTO-001 trial, the most common G3–4 adverse event was hypertension (14%). Other treatment-related CAEs were QTc prolongation (G3 3% and 1 case of G4 toxicity) and hypercholesterolemia (35%, G3 1.7%) [131]. In the full safety population of the study (*n* = 796) patients, hypertension was confirmed as the most common G3–4 treatment-emergent AEs (19.7%, any grade 41%) [132]. Dose interruption and dose reduction because of hypertension were reported in 6.3% and 1.3% of patients, respectively [132]. Similar incidence was confirmed in the phase III registrational trial (see Table 1) [74].

Real-world data derived from FAERS confirmed the previously reported data for hypertension but highlighted new possible CAEs, such as myocardial ischemic injury for pralsetinib and torsade de pointes/QT prolongation for selpercatinib [133,134].

The probable mechanism for RET inhibitors-related hypertension is the partial selectivity they have for VEGFR1 and 3, an off-target effect [130]. The VEGFR pathway causes hypertension by decreasing nitric oxide, with subsequent vasoconstriction [135].

In addition, these TKIs seem to upregulate CD47 (also via ERK feedback as seen in BRAF inhibitors), which is implicated with nitric oxide in the regulation of vasodilation and endothelial functions [135,136].

### 2.7. METex14 Inhibitors

MET transmembrane receptors, after homodimerization, activate many different intracellular signalling pathways (MAPK, PI3K-AKT, STAT and Nuclear Factor Kappa B [NF-κB]) leading to cell proliferation [137]. 

*MET* exon 14 skipping mutations account for ~2% of NSCLC with AGA. *MET* amplifications are also described in NSCLC but at present are not targetable [138]. For *MET*ex14 skipping mutated NSCLC, capmatinib and tepotinib both proved effective and are now approved.

Neither of these drugs showed relevant CAEs in the registration trials (GEOMETRY and VISION), except for one single episode of cardiac arrest that was deemed possibly related to capmatinib (see Table 1) [75,76,139,140].

In vivo experiments even suggested a possible, off-target, cardioprotective role of capmatinib, because it reduces ROS and pro-inflammatory molecules [141].

### 2.8. HER2 Inhibitors

The HER2 pathway and role in carcinogenesis have been widely studied in breast cancer when it is overexpressed and targeted by different effective monoclonal antibodies. These drugs are related to relevant and well-known cardiotoxicity, mainly HF and LVEF decrease [142]. However, in NSCLC the mechanism for HER2 overexpression is different and monoclonal antibodies have proven ineffective. 

Trastuzumab deruxtecan (Tdx) is an antibody–drug conjugate of the humanised anti-HER2 Immunoglobulin (Ig) G1 and the topoisomerase I inhibitor, and it is currently the only HER2-targeted drug approved for NSCLC.

Given the known cardiotoxicity of trastuzumab, patients with a known history of cardiac disease were excluded from all Tdx trials. 

In the FDA label pooled safety analysis of all patients that received Tdx in clinical trials at the recommended dose of 5.4 mg/kg, LVEF decrease was reported in 3.8% of patients (0.6% G3) [143,144]. In the trial for NSCLC (which selected patients for HER2 overexpression), DESTINY-LUNG-01, no CAEs were reported at the recommended dose of 5.4 mg/kg [77] (see Table 1). No data for cardiac safety are yet reported for the trial of mutated patients, DESTINY-LUNG-02.

In a comprehensive meta-analysis of 15 studies (1970 patients), the low incidence of decreased LVEF was confirmed (1.95%, 0.26% with HF), while QT interval prolongation was observed in 7.77% of cases [145].

The low incidence of LVEF decrease could be attributed to the different mechanism of Tdx from trastuzumab, as mentioned above.

### 2.9. NTRK Inhibitors

The NTRK pathway plays a fundamental role during embryogenesis and is altered in a great variety of tumours, though in a very small percentage (<1% in NSCLC) [146]. TKIs that target NTRK cancers obtained agnostic approval. Entrectinib and repotrectinib have already been described above due to their other indications.

Larotrectinib is the other drug approved for *NTRK* fusions. CAEs reported from a pooled analysis of the principal phase I–II trials (that comprehended 9% NSCLC) are reported in Table 1, since a dedicated trial for NSCLC has not been carried out due to the rarity of the mutation. 

Given the wide variety of patients included in these clinical trials, it is difficult to draw conclusions, but real-world data seem to confirm the low cardiotoxicity of these drugs [112].

## 3. Prevention and Treatment

### 3.1. Baseline Risk Evaluation

As described above, targeted therapies currently approved for the treatment of NSCLC are associated with a wide range of cardiotoxic effects. However, not all patients receiving these potentially cardiotoxic agents experience cardiotoxicity during the course of treatment, and specific subgroups at higher risk can and should be identified. Both therapy-related factors as well as patient-related parameters contribute to cardiovascular (CV) risk in oncological patients. 

Both lifestyle and concomitant medical conditions must be taken into account. For the former, particularly tobacco use, obesity, hypertension, diabetes, and hyperlipidemia should be considered [2,6,8,147]. For the latter, pre-existing CV disease, previous chemotherapy or thoracic radiotherapy must be evaluated [2,6,8], along with concomitant medications [7]. 

In a recently published retrospective real-world study (*n* = 451) analysing patients with NSCLC treated with TKI or immunotherapy, cardiotoxicity risk was increased with prior diabetes mellitus (OR 1.93, 95% CI, 1.04–3.61, *p* = 0.038), history of smoking (OR 1.91, 95% CI, 1.13–3.22, *p* = 0.016), and presence of baseline CV disease (OR 2.03, 95% CI, 1.13–3.64, *p* = 0.018) [2]. Combination of more than one factor had an additive or synergistic effect (smoking status + diabetes OR 3.03, 95% CI, 1.40–6.55, *p* < 0.01; smoking status + previous CV disease OR 1.99, 95% CI, 1.03–3.84, *p* = 0.041) [2]. Another retrospective cohort analysis (*n* = 123) of patients with advanced NSCLC treated with osimertinib noticed that severe cardiac AEs occurred primarily in patients with a history of CV risk factors or disease [23]. Multivariate analysis from a second trial which evaluated 58 patients by serial echocardiography before and after osimertinib administration confirmed that a history of heart disease was a significant predictor of cardiac dysfunction (ORR, 4.97; 95% CI, 1.26–19.6; *p* = 0.022) [148].

Of note, given the increasing use of immune checkpoint inhibitors (ICIs) in patients with early-stage NSCLC [149], prior immunotherapy treatment must also be taken into account. It is known that ICIs can have cardiotoxic effects, and recent reviews have reported a higher incidence of severe adverse events in patients who received TKIs after ICIs in NSCLC [150,151]. However, data are preliminary, and among the described cases, only a small percentage reported cardiotoxicity, primarily hypertension. 

Given the impact of all these clinical and demographic variables on the risk of developing cardiotoxicity, a baseline CV risk assessment in cancer patients prior to starting potentially cardiotoxic cancer treatments, including targeted therapies, is recommended. 

In 2020, a position statement from the Cardio-Oncology Study Group of the Heart Failure Association of the European Society of Cardiology in collaboration with the International Cardio-Oncology Society proposed new HFA-ICOS baseline CV risk stratification proformas [152]. Notably, only seven classes of drugs were considered (anthracyclines, HER2-targeted therapies, VEGFR inhibitors, BCR-ABL inhibitors, proteasome inhibitors and immunomodulatory drugs, RAF and MEK inhibitors, or androgen deprivation therapies), and the majority of targeted therapies currently used in NSCLC were not included; however, these proformas can be translated and applied in NSCLC patients, too.

A baseline CV evaluation including blood pressure measurement and ECG for QTc measurement should be performed in all patients [153,154]. QT interval is a surrogate marker for cardiac repolarization abnormalities, with significant prolongation associated with the development of potentially life-threatening ventricular arrhythmias; QTc interval should be calculated by Fridericia’s formula as it has demonstrated less error than other correction methods [154]. Combining these data with patients’ medical history and lifestyle helps stratify patients and identify them as at low, moderate, high, or very high risk of cardiotoxicity [152]. A baseline transthoracic echocardiogram (TTE) is advised for all high-risk patients with pre-existing CV comorbidities. A baseline quantitative evaluation of LVEF and diastolic function is needed to have a precedent, should symptoms suggestive of CV dysfunction occur during treatment [153,154]. In patients with pre-existing CV comorbidities, provocative testing should also be considered to rule out any residual coronary artery disease, and the severity of valvular diseases should also be assessed [152].

Additionally, measuring cardiac serum biomarkers (cardiac troponin (cTn) I or T, and natriuretic peptides (NP; BNP or NT pro-BNP)) is essential if ongoing monitoring during treatment is planned. Although routine use of these biomarkers is not well established, in selected situations they may also help to predict CV toxicities, particularly cardiomyopathy and/or HF, and therefore a baseline evaluation should be considered for high-risk patients [154]. Depending on the type and severity of the pre-existing CV disease, additional investigations—including resting or stress echocardiography, cardiac magnetic resonance, cardiac scintigraphy, and coronary computed tomography angiography—may be indicated to further assess the risk status.

Of note, baseline CV risk stratification should be completed promptly and should not delay the start of cancer treatment. 

The subsequent management of cardiotoxicity involves a multifaceted approach, including prevention, early detection, and treatment of possible CV complications (see Table 2).

### 3.2. Prevention Strategies

Patients identified to be at high or very high risk for cancer-treatment-related cardiotoxicity at baseline should be referred for a cardio-oncology or cardiology assessment to optimise the management of their pre-existing CV disease and modifiable CV risk factors. In addition, the pros and cons of the anticancer treatment should be discussed and a personalised management plan for surveillance during therapy should be provided. This could not only reduce the risk of morbidity and mortality, but also improve outcomes by reducing interruptions in cancer treatment due to CV events. Selected moderate-risk patients may also benefit from a cardio-oncology referral [153].

Prevention strategies with CV treatment interventions should be considered to mitigate CV risk for both patients without pre-existing CV comorbidities (primary prevention) and for those with prior or active CV disease (secondary prevention) [155].

Optimising modifiable CV risk factors is the first step of primary prevention, especially for those patients with extended life expectancies and/or those in whom anticancer treatment may be curative. This includes smoking cessation, limiting alcohol intake (max 100 g per week), and ensuring sufficient physical activity [156]. Exercise prescription appears to be a promising intervention, with various types of training tailored to the patient’s individual characteristics that can be incorporated during cancer therapy [157]. When baseline QTc prolongation is recognised (men > 440 ms, women > 450 ms), the correction of reversible causes (i.e., electrolyte imbalance, concomitant medications such as certain antibiotics or antiemetics) and the identification of genetic conditions that prolong QT are recommended. 

The addition of prophylactic pharmacological therapy is debated. ACE inhibitors (ACEi), beta-blockers (BB), and mineralocorticoid receptor antagonists (ARB) may be considered as they showed a significant benefit in preventing LVEF in patients treated with anti-HER2 therapy and chemotherapy [153]. Sacubitril/valsartan could be used in patients treated with crizotinib as it has been shown to limit most treatment-induced cardiotoxicities [158]. However, no statistical differences were seen in the incidence of overt HF or other clinical outcomes and it is not clear how these results can be translated to patients with NSCLC receiving targeted therapies. 

As there is recent evidence that hyperlipidaemia contributes to inflammation in patients with cancer [159], statin treatment may be beneficial.

### 3.3. Surveillance

Regular clinical evaluations, physical exams, and cardiovascular assessments are recommended for patients receiving cardiotoxic cancer therapies [153,154]. The frequency and modality of monitoring should be based on the patient’s baseline risk, the cardiotoxicity profile of the drug, and the emergence of any new CV symptoms. A summary related to the principal drug classes is reported in Table 2. Surveillance strategies may allow early detection of cardiotoxicity and therefore early intervention that has potentially life-saving implications.

A 12-lead ECG monitoring is required in patients at risk of cardiac arrhythmias or QT prolongation, according to specific drug protocols. It should be obtained once steady-state levels are achieved, if the dosage is increased, or with the development of an electrolyte imbalance [153,154].

NP and cTn could be used in monitoring. However, generally accepted cut-offs and reference values of these biomarkers have not been established yet in oncological patients, and levels may differ according to local laboratories or other factors (i.e., renal function, pulmonary embolism, infections). Therefore, an increase in biomarkers level should be interpreted in the patient’s clinical context [153,154].

TTE is the preferred technique to detect and confirm cardiac dysfunction. It should include a three-dimensional echocardiography and an LVEF measurement. An LVEF of 53% is considered a key metric to discern normal function vs. cardiac dysfunction and a reduction of LVEF of more than 10% from baseline to a value below the low normal limit suggests left ventricular dysfunction [160,161]. The incorporation of global systolic longitudinal myocardial strain (GLS) assessment is particularly important in patients with low-normal LVEF due to its higher sensitivity to subtle damage of the myocardial ultrastructure that would otherwise be undetectable by echocardiography [162,163]. A 10%–15% early reduction in GLS during treatment is considered the most reliable indicator for predicting cardiotoxicity [164]. Of note, American guidelines recommend monitoring left ventricular ejection fraction only in patients receiving anthracyclines, trastuzumab, or both [165], whereas European guidelines advise performing a resting echocardiogram in all patients undergoing potentially cardiotoxic treatments [153].

The use of cardiac magnetic resonance could be considered in patients with poor TTE image quality or when TTE is not diagnostic, but limitations in availability, cost and expertise may impede the wide adoption of this technique [166].

Notably, cancer survivors exposed to potentially cardiotoxic therapies should be monitored regularly over a long period, as some toxicities may occur several years after cancer treatment [153].

### 3.4. Treatment

When a CAE is identified, prompt intervention is crucial. A coordinated cardio-oncological multidisciplinary approach is recommended. The administered TT, type and grade of the CAE, and baseline patient CV risk should all be considered to select the optimal treatment and management. Interventions will depend on the severity of the condition and may involve dose adjustments, the introduction of cardioprotective medications, or, in some cases, discontinuation of the treatment.

#### 3.4.1. QTc Prolongation

A prolonged QT interval can cause ventricular tachycardia and Torsades de Pointes, with potentially fatal outcomes. 

In patients who develop QTc prolongation > 480 ms during TKI treatment, all possible contributing factors should be assessed and corrected, primarily drug-drug interactions and electrolyte imbalances. If the QTcF is 480 to 500 ms, weekly ECG monitoring is recommended but treatment could be continued [153,154].

If the QTc is ≥500 ms, the cancer therapy should be temporarily interrupted; an ECG should be repeated every 24 h until QTc prolongation resolves and inpatient admission for continuous cardiac monitoring should be considered, especially in patients with severe QTc prolongation (>550 ms). If the QTc remains >500 ms after correcting reversible causes, an alternative anticancer treatment should be discussed. For the other patients, treatment restarting should be considered, with the same or lower dose according to drug-specific protocols [153,154]. Subsequent weekly ECG monitoring during the first 4–6 weeks and then monthly thereafter is recommended after restarting QTc-prolonging cancer therapy [153,154].

Although there are no recommendations, in cases of QTc prolongation associated with severe bradycardia or sinus pauses, isoprenaline infusion or temporary pacing could be beneficial [153].

TKI treatment should be permanently discontinued in patients who develop Torsades de Pointes or sustained ventricular tachyarrhythmia [153,154].

#### 3.4.2. Hypertension (HTN)

HTN is common in oncological patients, but it is often linked to a pre-existing condition rather than the anti-cancer treatment itself. However, pre-existing hypertension combined with increasing age is the main risk factor for developing TKI-induced HTN during TKI treatment [167]. 

Before and during treatment, the systolic blood pressure (BP) should be consistently <140 mmHg and the diastolic blood pressure < 90 mmHg. In patients who develop HTN during TT, it is recommended to assess and correct possible contributing factors (i.e., stress, pain, excess alcohol consumption, renal impairment, sedentary lifestyle, and obesity) before considering the interruption of cancer treatment [153].

Pharmacological treatment with ACE-inhibitors or ARBs is recommended as first-line therapy to reduce the risk of cancer-therapy-related cardiac dysfunction; dihydropyridine calcium channel blockers could be used as second-line antihypertensive drugs [153,154]. The combination of ACE-inhibitors/angiotensin receptor blockers and dihydropyridine calcium channel blockers is preferred with systolic BP ≥ 160 mmHg and diastolic BP ≥ 100 mmHg. 

If severe HTN is diagnosed (systolic BP ≥ 180 mmHg or diastolic BP ≥ 110 mmHg), any cancer therapy associated with HTN should be deferred or temporarily withheld until the BP is controlled to values (160/100 mmHg). Cancer therapy can be restarted once BP is controlled, with consideration for dose reduction. 

Other drugs such as transdermal nitrates, beta-blockers, and diuretics may be considered according to the underlying condition in treating HTN [153].

#### 3.4.3. Symptomatic or Asymptomatic CTRCD

Early treatment of symptomatic and asymptomatic severe cancer-therapy-related cardiac dysfunction (CTRCD, defined as LVEF < 40%) is crucial to prevent worsening HF. A multidisciplinary approach is recommended to guide clinical decisions and hospitalising the patient could be necessary. 

Both moderate to severe asymptomatic CTRCD and moderate to severe symptomatic CTRCD require temporary interruption of the TT and rapid initiation of HF medication (combinations of ACEi or ARB plus BB and spironolactone). Intravenous or oral diuretics should be used in cases of congestion. Sacubitril-valsartan should be considered in place of the ACEi or ARB if LVEF has reduced to <40% [153,154].

Re-exposure to TT should be discussed after recovery of cardiac function, depending on the severity of the left ventricular systolic dysfunction, HF syndrome, the intensity of medication required to restore normal function, pre-existing other CV comorbidities, and treatment alternatives [167].

In mild asymptomatic CTRCD—asymptomatic patients with normal LVEF but a decrease in average GLS from baseline assessment (12% relative decrease or 5% absolute decrease) and/or rise in cardiac biomarkers—initiation of cardioprotective treatments should be considered if not already administered. Repeat LVEF/strain measurement every 3 months is then required [154].

#### 3.4.4. Hyperlipidemia

Hyperlipidemia, including hypercholesterolemia and hypertriglyceridemia, in most cases, is easily managed with appropriate medical therapy. Lipid-lowering agents are recommended at the first sign of elevated cholesterol and/or triglyceride levels [95]. Rosuvastatin and pravastatin are usually preferred due to their low involvement with CYP450 enzymes [168]. The addition of fibrates or fish oils could be considered for lowering triglyceride levels if treatment beyond statins is required [153,154]. Ezetimibe or a combination of statins and ezetimibe are other effective options. 

In cases of severe hyperlipidemic events (cholesterol levels > 500 mg/dL and/or triglyceride levels > 1000 mg/dL) despite the use of lipid-lowering therapies, dose interruption is recommended. In case of recurrence, a dose reduction is suggested [153,154]. 

Dietary intervention and physical activity should also be promoted to control hyperlipidemia.

## 4. Conclusions

Targeted therapies in NSCLC are associated with varying grades of cardiotoxicity, some of which are still not fully comprehended. Every drug class has its own typical potential profile, but real-world data show that even if rare, CAEs must not be underestimated and must always be considered when treating our patients. Fortunately, cardio-oncology societal guidelines are available nowadays to assist the clinician. However, a multidisciplinary approach for baseline evaluation, monitoring and treatment of cardiovascular diseases in oncologic patients must be implemented in daily practice. In addition, dedicated subanalyses or dedicated real-world studies remain fundamental to better understanding the potential cardiotoxicity of these new therapies, to preserve the survival and clinical benefit that these drugs provide.

## Figures and Tables

**Table 2:** Cardiotoxicity profile and management of principal targeted therapies in NSCLC.

Drug Class	Cardiotoxicity Profile	Baseline Evaluation	Surveillance	Management
EGFR inhibitors	QT prolongation, HF	ECG, BP, TTE, blood test for electrolytes	Serial ECG. Three-monthly TTE should be considered. Close monitoring of electrolytes (especially potassium and magnesium levels).	Correct electrolytes, avoid/suspend QT-prolonging concomitant medications
ALK/ROS1 inhibitors	HTN, hyperglycaemia, dyslipidemia, sinus bradycardia and AV block, QTc prolongation	ECG, BP, blood test for cholesterol levels and glycaemia	ECG 4 weeks after the start of treatment and every 3–6 months thereafter. BP monitoring. Cholesterol levels and glycaemia at first follow-up visit and every 3–6 months	Avoid nodal blocking agents, lipid-lowering agents (statin and ezetimibe)
BRAF + MEK inhibitors	HTN; HF/left ventricular dysfunction; QT prolongation	ECG, BP, TTE	Clinical exam to screen signs of HF. BP measurement weekly for the first month and then every 3 months. ECG every 3 months	ACE inhibitors/angiotensin receptor blockers; dihydropyridine calcium-channel blockers
RET inhibitors	HTN	ECG, BP, TTE	BP close monitoring (home diary). NP and TTE every 3 months	ACE inhibitors/angiotensin receptor blockers; dihydropyridine calcium-channel blockers
T-Dxd	HF	ECG, BP, TTE. Consider NP	TTE every 3 months. Clinical examination to check HF signs. In high risk patients consider NP monitoring	Consider protecting medications

Abbreviations: HF: heart failure, HTN: hypertension, BP: blood pressure, TTE: transthoracic echocardiography, NP: natriuretic peptides.
